# Electron paramagnetic resonance (EPR) meets drug delivery and biomaterials: a magnetic love story

**DOI:** 10.1007/s13346-025-01992-9

**Published:** 2025-10-15

**Authors:** Bernard Gallez

**Affiliations:** Louvain Drug Research Institute, Biomedical Magnetic Resonance, UCLouvain, Avenue Mounier 73.08, Brussels, B-1200 Belgium

**Keywords:** EPR, ESR, Drug delivery, Drug release, Biocompatibility, Oxygen

## Abstract

This narrative review underscores the powerful role of Electron Paramagnetic Resonance (EPR), also known as Electron Spin Resonance (ESR), in characterizing drug delivery systems (DDSs). Using drugs or probes tagged with spin labels, EPR provides detailed insights into structural and dynamic properties, as well as the molecular microenvironment (including micro-viscosity, micro-polarity, and micro-pH) and enables real-time monitoring of drug release and degradation processes both in vitro and in vivo. In nanomedicine research, EPR can also serve as a quantitative tool to track the fate of DDSs doped with iron oxide particles that are used in theranostics. Beyond DDS characterization, EPR has contributed substantially to elucidating radical mechanisms within material matrices, notably in bone cements and dental resins used for restorative applications. Moreover, incorporating paramagnetic compounds into DDSs or biomaterials has broadened the scope of EPR applications, enabling precise measurements of oxygen and nitric oxide levels in complex biological environments. The incorporation of oxygen sensors into biocompatible matrices has also enabled the development of implantable resonators for measuring oxygen at substantial tissue depths. Incorporating oxygen sensors into cell therapy implantable devices or grafted tissues can serve as an indicator of both oxygenation and vascularization.

## Graphical abstract

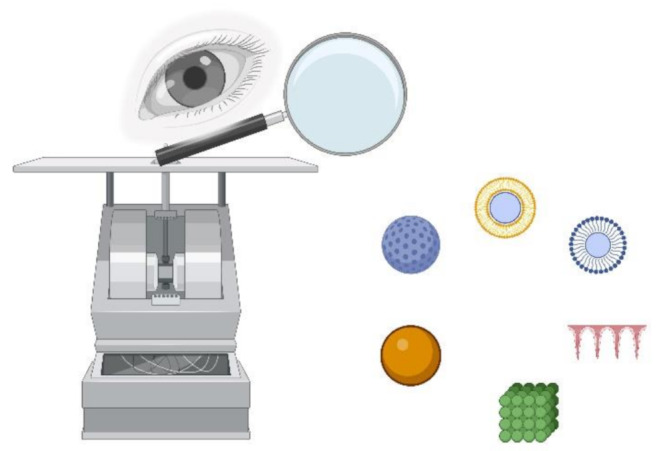

## Introduction: EPR in biomedical research

Electron Paramagnetic Resonance (EPR), also known as Electron Spin Resonance (ESR), is a magnetic resonance technique that selectively detects species containing unpaired electrons by measuring the absorption of microwave radiation in the presence of an external magnetic field. EPR has a wide range of applications across various disciplines, including chemistry and photochemistry (e.g., studying radical reactions and characterizing metal complexes), biochemistry (e.g., investigating paramagnetic catalytic centers in enzymes), and biology and toxicology (e.g., identifying reactive radical species). Notably, EPR has also found significant applications in the pharmaceutical sciences [1–4]. This review focuses on drug delivery systems (DDSs), aiming to illustrate the utility of EPR in their characterization, as well as to demonstrate how the use of suitable DDSs or biomaterials have expanded the applications of EPR spectroscopy and imaging in biology and medicine.

### Principle of EPR – Parameters relevant to drug delivery and biomaterials

It is likely that many readers of this review come from a pharmaceutical background and may have limited familiarity with quantum mechanics. Unfortunately, most EPR textbooks begin with the so-called spin Hamiltonian and an introduction to complex, lengthy equations, which can pose a significant barrier to understanding (despite their appeal to physicists and physical chemists). Therefore, for readers without a strong background in physics, such as biologists and pharmacists (myself included), this section aims to simplify the concepts by presenting the basic principles of EPR and highlighting key parameters, thereby providing a foundation for interpreting EPR data [5].

**Fig. 1:**
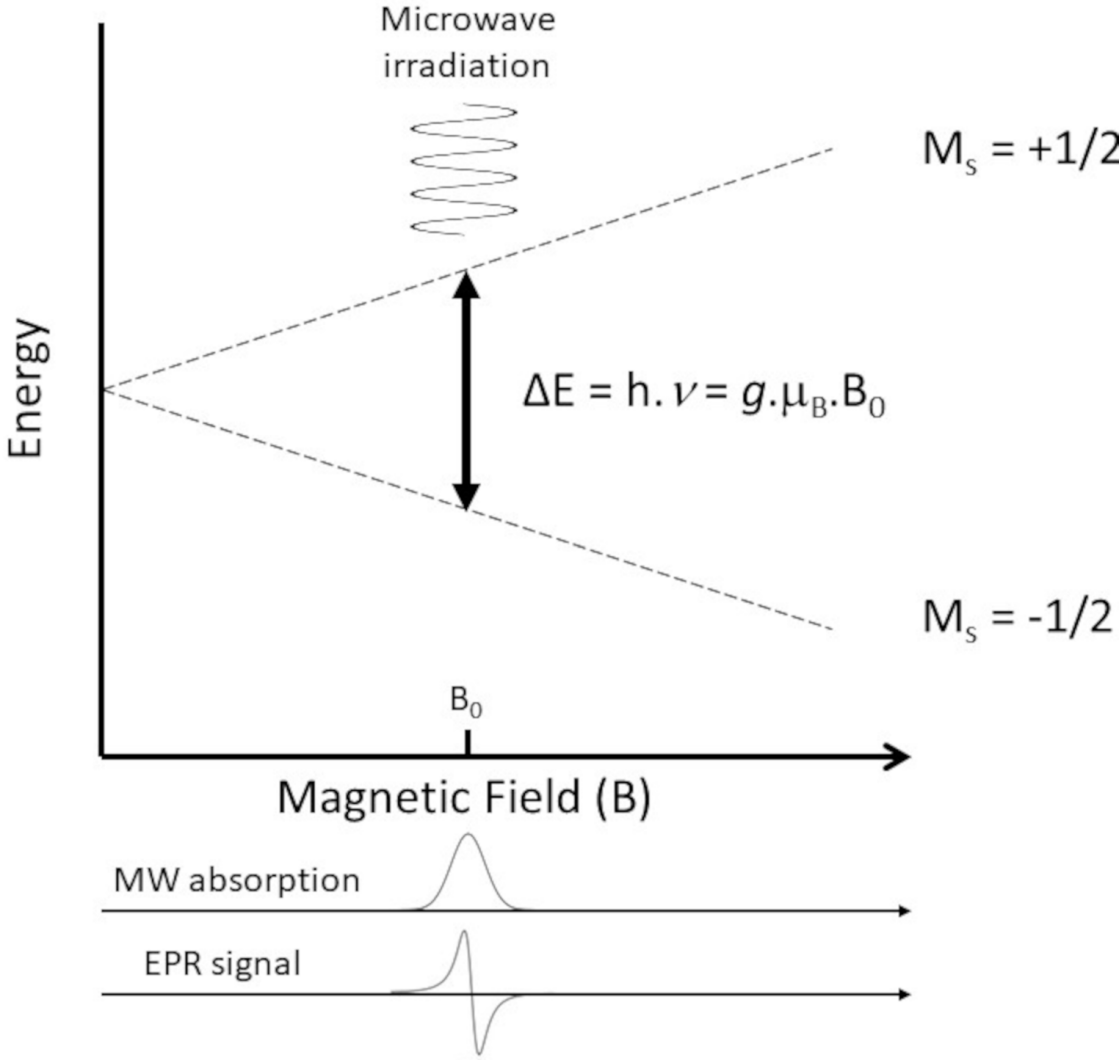
Fundamental principle of EPR. The principle of continuous-wave (CW)-EPR involves continuously irradiating a sample with microwaves at a fixed frequency while gradually sweeping the external magnetic field. Unpaired electron spins in the sample absorb microwave energy at specific magnetic field strengths where their energy levels match the microwave frequency. At the resonance, the energy difference (ΔE) between these two states, that is proportional to the strength of the applied magnetic field (B_0_), satisfies the following equation: Δ E = h.*ν* = *g*.µ_B_.B_0_ where h is Planck’s constant, *ν* is the frequency of the electromagnetic radiation, *g* is a constant termed *g* factor, and µ_B_ is the Bohr magneton

**Fig. 2:**
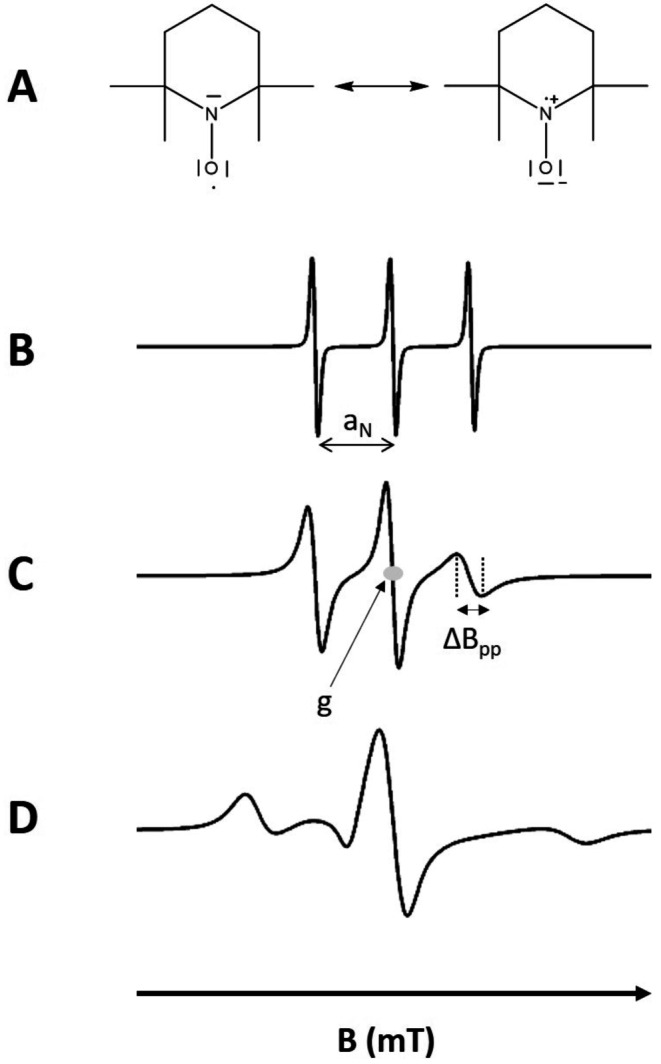
Typical EPR spectra of nitroxide radicals. (**A**) chemical structure of TEMPO, a six-membered ring nitroxide. The unpaired electron is delocalized on the oxygen and the nitrogen atoms. (**B**) EPR spectrum of a freely tumbling nitroxide in low viscosity medium. The hyperfine splitting a_N_ is visualized on the spectrum. (**C**) EPR spectrum of nitroxide in a more viscous solvent. Note the line broadening and the presence of asymmetry in the EPR spectrum with a high-field line with decreased amplitude. The g value and the peak-to-peak linewidth (ΔB_pp_) are represented on the spectrum. (**D**) powder spectrum of a nitroxide in a solid state. (adapted from ref [6])

The principle of EPR is similar to the more familiar nuclear magnetic resonance (NMR). Both methods are based on the interaction of electromagnetic radiation with magnetic moments caused by electrons (EPR) or nuclei (NMR). When placed in an external magnetic field, the magnetic moment of an unpaired electron will align parallel or antiparallel in the direction of the magnetic field, which corresponds to a lower (Ms = −½) or an upper (Ms = +½) energy state. The principle of continuous-wave (CW)-EPR involves continuously irradiating a sample with microwaves at a fixed frequency while gradually sweeping the external magnetic field. Unpaired electron spins in the sample absorb microwave energy at specific magnetic field strengths where their energy levels match the microwave frequency (Fig. 1). At the resonance, the energy difference (ΔE) between these two states, that is proportional to the strength of the applied magnetic field (B_0_), satisfies the following equation:

$$\Delta \,E = h.v = g.{\mu _B}.{B_0}$$

where h is Planck’s constant, *ν* is the frequency of the electromagnetic radiation, *g* is a constant termed *g* factor (g = 2.0023 for an unpaired electron), and µ_B_ is the Bohr magneton. The *g*-factor is a dimensionless parameter that characterizes the magnetic moment of an unpaired electron in a paramagnetic substance. It measures the splitting of an electron’s energy levels under the influence of an external magnetic field and it provides information on the electronic environment of the unpaired electron allowing the identification of a particular radical species. It is derived from the position of the EPR line in the EPR spectrum. For those familiar with NMR spectroscopy, it is rather comparable to the NMR chemical shift. The unpaired electron may interact with neighboring nuclei (with nuclear spin *I* different from 0) to produce splittings in the EPR spectrum, that are called hyperfine splittings (hfs). The type and number of the nuclei interacting with the electron determine the number of lines and their relative intensities, according to the rule:

$$Number{\rm{ }} of{\rm{ }} lines{\rm{ }} = {\rm{ }}2.n.I + {\rm{ }}1$$

where *n* is the number of coupling nuclei and *I* is the nuclear spin. Electron-nuclear hyperfine coupling can be compared to the nuclear-nuclear spin-spin coupling in NMR. For example, magnetic interaction between the free electron and the nuclear spin of nitrogen (*I* = 1, for the stable isotope ^14^N) in nitroxides results in a hyperfine splitting into three lines (Fig. 2A-B). For the methyl radical CH_3_°, there is a coupling with 3 hydrogens (I = ½) leading to 4 lines in the EPR spectrum (the coupling with C can be neglected as the most abundant isotope is ^12^C with *I* = 0). The hyperfine splitting constant is labelled by the symbol a followed by a subscript indicating the splitting nucleus (a_N_ in the example of a nitroxide, Fig. 2). Other parameters easily derived from the EPR spectra are the peak-to-peak linewidth (ΔB_pp_) and the signal amplitude of the peaks (I). As EPR spectra are recorded usually in the form of the first derivative, the signal intensity I can be calculated by the double integration of the EPR spectra. The different parameters are illustrated in the EPR spectrum of a nitroxide (or aminoxyl radical), a commonly used paramagnetic compound in EPR spectroscopy (Fig. 2). As illustrated in the coming paragraphs, the shape of an EPR spectrum and its parameters are strongly dependent on the microenvironment of the unpaired electron present in the paramagnetic sensor introduced in the studied medium (for example, a DDS).

A first example is the dependence on the polarity of the medium. Let’s consider the case of a nitroxide where the signal is split into three lines due to interactions between the unpaired electron and the nitrogen atom it’s attached to. The polarity of the medium affects how tightly the unpaired electron interacts with the nitrogen atom. In polar solvents (like water), the electrons are more localized around the nitroxide group. This increases the interaction between the electron and the nitrogen nucleus, making the hyperfine splitting constant a_N_ larger. Conversely, in less polar environments, the electron cloud is a bit more spread out or delocalized. This reduces the interaction with the nitrogen nucleus, so the hyperfine splitting constant gets smaller.

So far, we have considered liquid spectra of rapidly tumbling free radicals which give isotropic hyperfine splittings and average *g*-factors. In DDSs, important changes in micro-viscosity may occur during the release processes that are important to characterize. If we are considering the case of a rapidly tumbling nitroxide in solution, the EPR spectrum will be characterized by three lines with almost equivalent intensities and narrow linewidths (Fig. 1B). If the micro-viscosity increases, the tumbling rate will decrease leading to a line broadening and the presence of asymmetry in the EPR spectrum with a high-field line with decreased amplitude (Fig. 2C) [3, 5, 6]. This asymmetry of the spectrum will continue to increase with the increasing rigidification of the system (as for solid-state spectra recorded at cryogenic temperatures) (Fig. 2D) [6]. The use of simplified equations or spectral fitting gives access to an estimation of the rotational correlation time τ_c_.

**Fig. 3:**
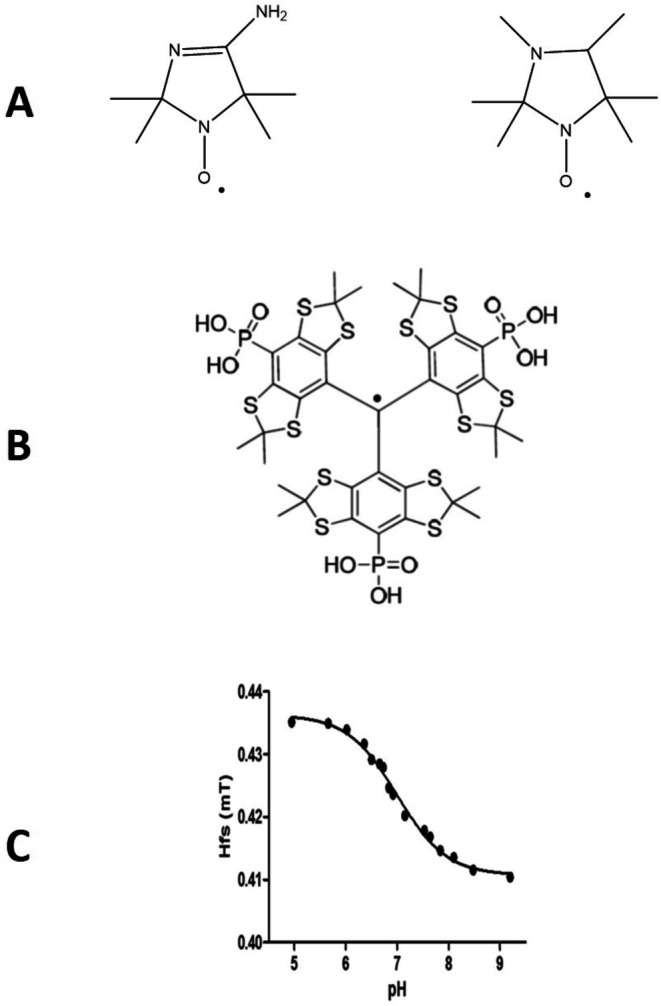
Paramagnetic probes suitable for pH measurements. (**A**) pH-sensitive nitroxides. Left : 4-amino-2,2,5,5-tetramethyl-3-imidazolidine-1-oxyl. Right : 2,2,3,4,5,5-hexamethyl-imidazoline-1-oxyl. (**B**) pH-sensitive trityl, Tris(8-phosphono-2,2,6,6-tetramethylbenzo[1,2-d;4,5d’]bis [1, 3]dithiol-4-yl)methyl. (**C**) calibration of the hyperfine splitting (hfs) measured as the distance between the two high-field components of the quartet of the compound presented in B in the pH range around 7. (adapted from ref [10])

One particularly relevant parameter for the study of DDSs is the micro-acidity. For this purpose, it is necessary to use paramagnetic sensors whose EPR spectrum is particularly sensitive to pH variations. This can be achieved using nitroxides that contain an additional amino group in their structures, such as imidazoline nitroxides (Fig. 3A) [2, 3, 7, 8]. The protonation of the amino group will lead to a decrease in spin density at the nitrogen of the nitroxide group resulting in a decrease in the hyperfine splitting a_N_. Another class of paramagnetic compound that can be used for the purpose is found among stable triarylmethyl (trityl) radicals, for example using phosphonated triarylmethyl radicals (Fig. 3B) [9–11]. The hyperfine splitting can be calibrated as a function of pH for a given nitroxide or trityl radical with the highest variation of hfs in the region of the pKa (Fig. 3C) [2, 3, 7, 8]. Interestingly, there is a wide variety of paramagnetic probes with different pKa values, enabling coverage of the full pH range and suitability for applications at any pH [3].

The examples described so far present spectroscopic applications of EPR which report a global information on the paramagnetic compounds over the entire volume. It does not provide information regarding the spatial distribution of spins within the object. To get additional spatial information, there is a need to perform EPR imaging (EPRI) [12]. EPRI utilizes magnetic field gradients in a manner similar to (nuclear) magnetic resonance imaging (MRI). EPRI can be used in purely spatial domain to obtain one, two- or three-dimensional images of free radical distribution [12]. To get information on the heterogeneity of spectra in different parts of the images, a fourth dimension is required which is called spectral-spatial imaging. This provides unique information of the EPR spectral shape in each voxel of the image and gives access to a unique information on the heterogenous distribution of polarity, pH or viscosity within a DDS or a biomaterial [3].

One final important consideration is the type of instrumentation to be used depending on the type of application [13]. The greatest difference between NMR and EPR arises because the gyromagnetic ratio of an unpaired electron is much larger than that of a proton, so the resonance frequency/magnetic field ratio for the electron is 28 GHz/T while it is 42.5 MHz/T for the proton. Consequently, standard EPR spectrometers or imagers operate at much higher frequencies and lower fields than conventional NMR spectrometers. Most standard commercial spectrometers operate at 9 GHz (also called X-Band) for a magnetic field sweeping around 0.34 T. At this frequency, non-resonant absorption of the electromagnetic radiation by the liquid water of the biological samples presents a serious problem, thus limiting the sample size to a thickness of less than 1 mm. Studies on larger aqueous samples or in vivo in animals can be studied only by reducing the operating frequency to 1 GHz or less. At 1 GHz, the depth of sensitivity is about 1 cm, which is ideal for studies in rodents [14]. The use of lower frequencies (300 MHz) would extend the sensitive depth to sampling volumes comparable to NMR studies [15]. Of course, reducing the operating frequency also significantly reduces detection sensitivity, which poses additional challenges when applying EPR in vivo.

### Applications of EPR in drug delivery systems

EPR spectroscopy and imaging have been used in many studies to characterize DDSs and understand fundamental mechanisms involved in drug release [3, 4]. The purpose here is more an illustration of capabilities rather than an exhaustive list of advanced applications. This paper also provides an opportunity to acknowledge Karsten Mäder’s pioneering contribution to this field of research. EPR spectroscopy and imaging have been used to characterize a wide range of systems, including hydrogels [16–19], microspheres [20–22], implants [8, 23–30], multilayer tablets [31, 32], coated tablets/pellets [32, 33], liposomes [34–42], nanoemulsions (31–32) [43–44], solid lipid nanoparticles and nanostructured lipid carriers [45–49], dendritic core-multishell nanocarriers [50–52], micelles [19, 35, 53–57], supramolecular nanostructures [58], and electrospun fiber sponges [59].

Key processes in controlled release include water penetration into the matrix, drug solubilization, drug diffusion, matrix degradation and/or carrier erosion [2]. As previously described, EPR spectra are highly sensitive to the motion of paramagnetic probes such as nitroxides. This feature could be exploited using spin-labeled compounds incorporated in DDSs. When immobilized in a solid matrix, the EPR spectrum has feature that looks to a powder spectrum (Fig. 1D). When solubilized, the nitroxide will display an EPR spectrum characteristic of more rapidly tumbling species. This unique capability to discriminate between species with different mobility can be exploited to characterize different mechanisms of release [2, 3]. In matrix erosion systems, drug release and matrix erosion occur simultaneously. In such devices doped with a nitroxide reporter, a typical solid state EPR spectrum will be recorded. During the release process, the shape of the EPR spectrum will not change over time, only the signal intensity will be reduced over time [29, 30]. Conversely, in diffusion-controlled release systems, water penetration will occur rapidly leading to the solubilization of the drug prior to its release. In such systems, a powder spectrum is observed in the starting material. As solubilization occurs, a mobile component will appear in the EPR spectrum. Actually, both mobile and immobile parts will be superimposed in the EPR spectra with the mobile part increasing over time [8]. Of note, this dynamic change can be monitored using simple nitroxides integrated into the DDS or using spin-labeled drugs. It allows for monitoring the kinetics and mechanisms of drug release in vitro and in vivo in a non-invasive and real-time manner. Studies have specifically used EPR to analyze the release of spin-labeled naproxen [16, 17, 60, 61], spin-labeled fatty acids [17], spin-labeled insulin [18], spin-labeled albumin [20, 22], spin-labeled paullone [62], and spin-labeled dexamethasone [48, 50, 51, 63].

In drug delivery systems, polarity is a crucial parameter because it dictates the interactions between the drug, the carrier material, and the surrounding medium. For example, in polymeric nanoparticles or implants, drug-polymer polarity compatibility controls how strongly the drug is bound within the matrix, affecting diffusion rates and degradation-triggered release. By matching or mismatching polarity between drug and carrier, it is possible to fine-tune whether release is rapid, sustained, or triggered by specific physiological conditions. Information regarding the localization of the probe or spin-labeled drug can be estimated by means of the hyperfine splitting constant. Important information regarding localization (and re-localization) of the drugs during DDSs formation or degradation have been obtained using EPR spectroscopy [35, 44, 48, 49, 51, 54, 56–58, 64, 65]. Besides solvent interactions, EPR also allows for studying interactions between drugs, carriers, and biological components (like cell membranes or proteins). For instance, EPR has been used to characterize interactions between spin-labeled drugs and bovine serum (BSA) or human serum albumin (HSA) [16, 17, 61, 63], dendrimers and model membranes [53], liposomes and stratum corneum [38], or the impact of drugs on the fluidity of lipid membranes [34, 36, 37, 39, 40, 47, 64]. EPR studies have also provided insight into how a model drug partitions within solid lipid nanoparticles and nanostructured lipid carriers [45–48].

Another important contribution of EPR to the study of DDSs is its ability to provide information on the acidic conditions inside the delivery systems during the release process. Polymeric systems made from polyanhydrides or polyesters may contain acidic monomers or release these monomers during their hydrolysis. This may lead to acidification that may affect the stability of drugs included in the DDSs. A large series of studies benefit of using EPR for tracking the dynamic changes in pH over time during the release process [8, 18, 19, 28, 50, 60, 61, 63, 66]. For example, it has been used to study internal pH in biodegradable microspheres (8) [21], multilayer tablets [31, 66], or BSA hydrogels [60, 61].

Low-frequency EPR spectrometers have enabled the non-invasive use of EPR in vivo in small animals to monitor the internal changes occurring in DDSs [8, 26, 27, 29, 30, 42, 66]. This allows for monitoring drug release and polymer degradation [8, 26, 27], implant formation [26, 27], thus offering a possibility for direct in vitro/in vivo correlation [26, 27, 29, 30]. In addition to spectroscopic information, EPR imaging allows obtaining the spatial distribution of paramagnetic species or microenvironmental conditions within delivery systems [3, 30, 32, 42, 66]. For example, it has been used to monitor internal pH gradients in multilayer tablets [32, 66] or to study the transport of liposomes in the skin [42, 62]. Spin labels with different isotopes (e.g., ¹⁵N vs. ¹⁴N) can be used in conjunction with EPRI to attribute mobility to specific layers within an implant [30].

### Role of EPR in theranostics

Another field of application where EPR may contribute to nanomedicine research lies in its use as a quantification tool to monitor the fate of DDSs doped with iron oxide particles. This falls within the field of theranostics, where DDSs can not only deliver a therapeutic agent but also serve as a means of preselecting patients based on non-invasive imaging data assessing the accumulation of the DDS at the target site [67]. While not limited to oncology, this area has been particularly active in recent years, with efforts focused on optimizing drug delivery to solid tumors. Superparamagnetic iron oxide (SPIO) particles are MRI contrast agents that predominantly induce T_2_/T_2_* relaxation effects. In addition, SPIO-loaded systems have been proposed for magnetic hyperthermia following exposure to time-varying magnetic fields [68] or as vectors for magnetic targeting [69]. While MRI can visualize the sites of SPIO accumulation, direct quantification of the actual amount delivered remains challenging due to the complex relationships between contrast effects, tissue relaxation properties, and iron oxide content. In preclinical studies, EPR is especially valuable for quantifying iron oxide concentrations in tissues. Iron oxide particles exhibit an EPR spectrum that directly reflects the number of particles in a sample, and this spectrum differs from those of other iron species [70, 71]. EPR has been used as a sensitive quantification method to assess the delivery of SPIOs functionalized with peptides that modulate biodistribution [70, 72], SPIO-labeled cells [71, 73, 74], PLGA nanoparticles [69, 75, 76], and magnetoliposomes [77].

### Contribution of EPR to biomedical materials science

Shifting from DDSs to biomaterials, EPR has also made significant contributions to understanding radical mechanisms occurring within material matrices and their associated properties. One example is the application of EPR to monitor molecular processes in polymethylmethacrylate (PMMA) bone cements [78, 79]. The concentration of free radicals generated during the polymerization of bone cement is sufficiently high to enable EPR monitoring of both the polymerization and curing processes. Since the presence of free radicals reflects the amount of unpolymerized chains in the polymer, this parameter can be linked to mechanical properties such as strength and density. For instance, free radical generation has been correlated with the polymer powder-to-liquid monomer ratio and initiator concentration, both of which directly affect polymerization kinetics [80]. Initial temperature before mixing the bone cement components has also been shown to influence free radical decay, consistent with changes in porosity [81]. Comparisons of mechanical properties and free radical concentrations after polymerization in vitro and ex vivo (following implantation in the canine femoral intramedullary cavity) revealed that in vivo curing (free radical disappearance) takes considerably longer (over 4 weeks) than in vitro curing (less than 2 weeks) [82]. Direct, noninvasive observation of free radicals produced during PMMA bone cement formation has been achieved in vivo using low-frequency EPR spectrometers [83]. These measurements, conducted over several days on the same animals, demonstrated that radical decay rates were significantly lower in vivo compared to in vitro conditions [83].

EPR has also been applied to study dental resin-based composites [84–90], which are primarily composed of inorganic fillers dispersed in an organic matrix of various dimethacrylate monomers combined with a photo-initiating system. During tooth restoration, the resin is placed into the cavity and photopolymerized in situ using a light-curing device emitting blue light. This photopolymerization process produces free radicals that initiate a chain reaction linking the dimethacrylate monomers into a three-dimensional polymer network. Two types of free radicals become trapped in this network: an allylic radical and a so-called propagating radical, each with distinct EPR patterns [84]. EPR has provided unique insights into the influence of storage conditions and irradiation modes on mechanical properties in relation to the polymerization process [85, 86]. Due to the limited curing depth, large or deep cavities must be restored in successive layers [87]. EPR imaging and spectral-spatial EPR imaging have been used to estimate allylic and propagating radical concentrations as a function of curing depth, revealing a strong dependence on illumination procedures [87, 88].

**Fig. 4:**
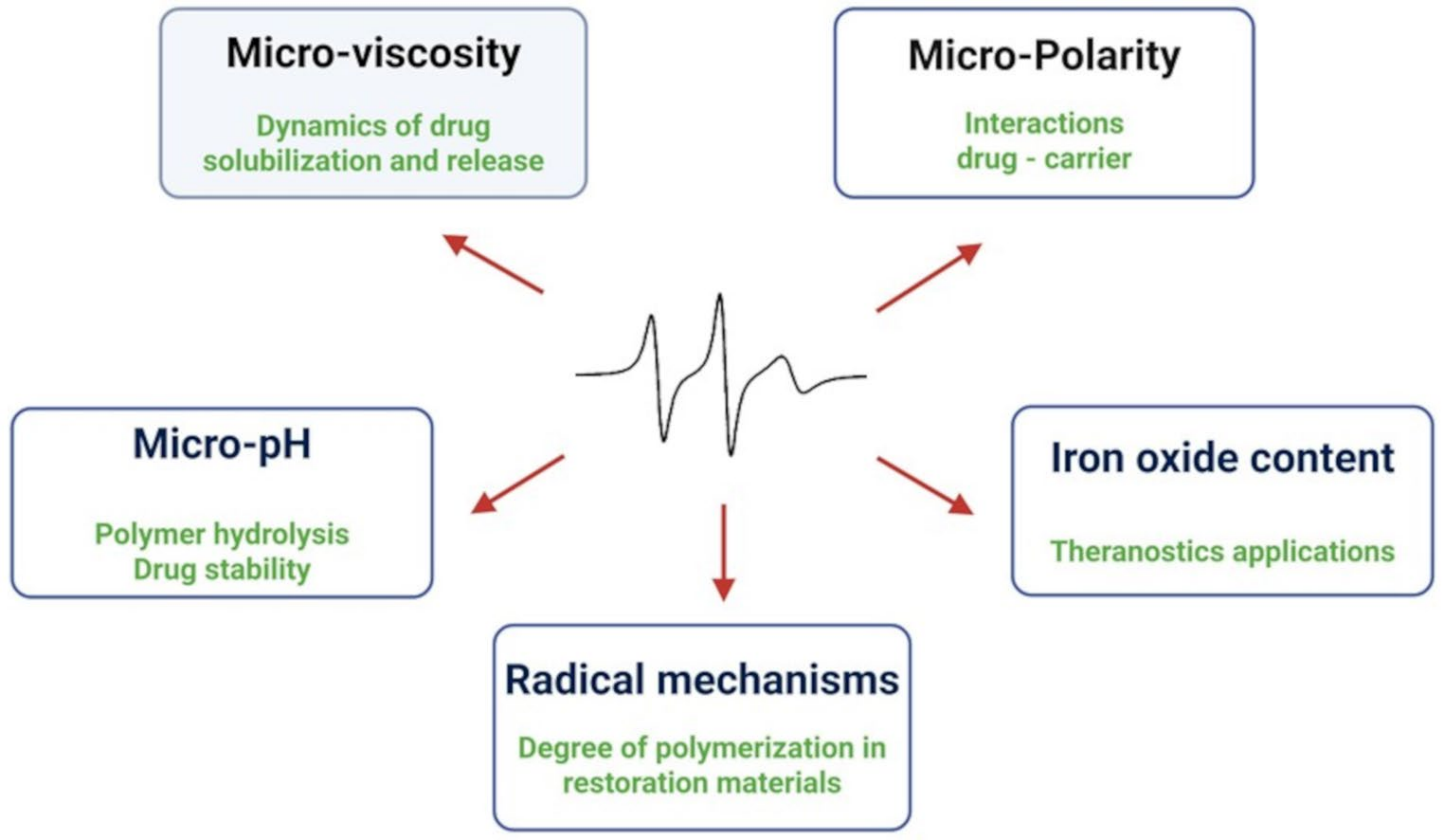
Key parameters provided by the analysis of the EPR spectra in DDSs and biomaterials. EPR provides on micro-viscosity (related to dynamics of drug solubilization and release), micro-polarity (related to the interactions between the drug and the carrier), and micro-pH (related to degradation of some polymers and influencing drug stability). EPR can also serve as a quantitative tool to track the fate of DDSs doped with iron oxide particles that are used in theranostics. EPR also elucidates radical mechanisms within material matrices, notably in bone cements and dental resins used for restorative applications

The key parameters that are extracted from the analysis of EPR spectra to characterize DDSs and biomaterials are summarized in Fig. 4.

### Drug delivery systems and biomaterials to expand EPR applications in oxygen and nitric oxide sensing

So far, we have discussed how EPR can provide valuable insights for research on delivery systems. We will now examine how DDSs and biomaterials have, in turn, contributed to expanding the biomedical applications of EPR, particularly in the measurement of oxygen and nitric oxide in tissues.

**Fig. 5:**
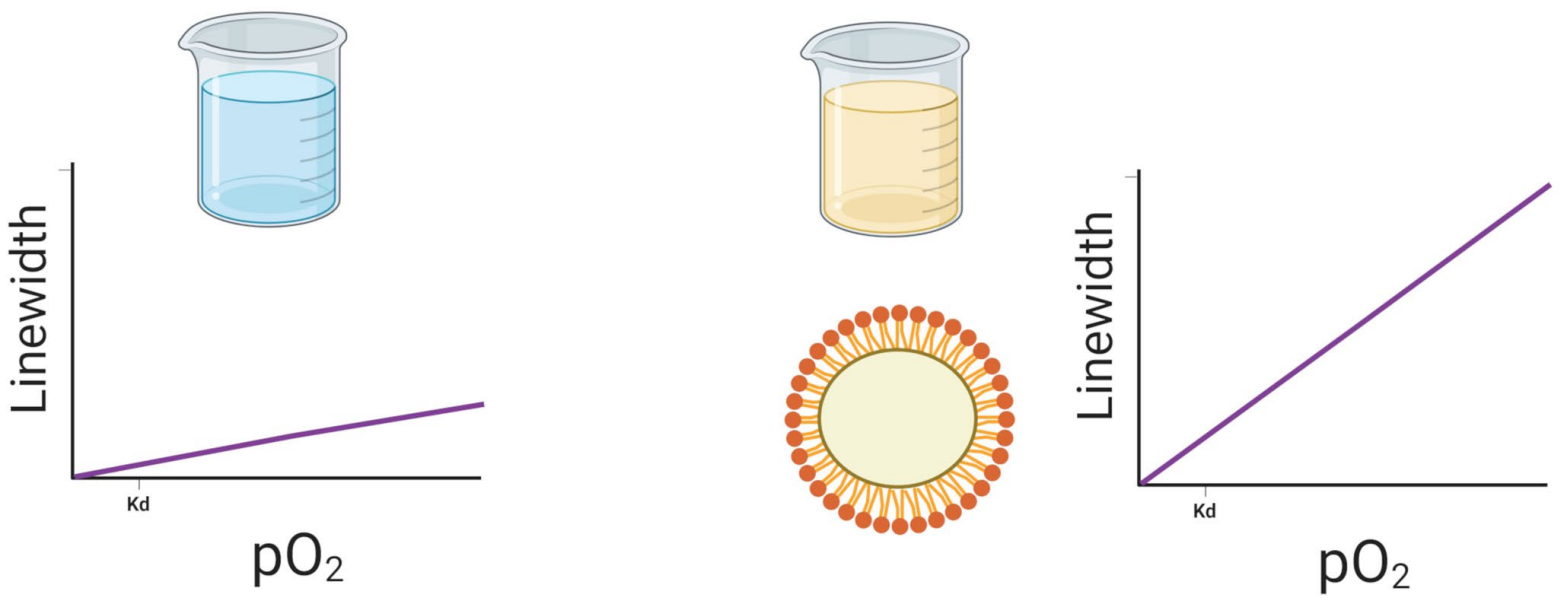
Benefits from the encapsulation of soluble EPR oxygen sensors in lipophilic solvents. The variation of EPR linewidth as a function of pO_2_ is fairly modest in aqueous solutions. Solubilization in an organic solvent increases the sensitivity of the probe as oxygen is much more soluble in lipophilic environments than in water. In other words, for a same change in pO_2_, the variation in oxygen concentration will be higher in lipid solvent leading a larger variation in EPR linewidth. A similar increase in sensitivity has been obtained using nano-objects with a lipophilic core

EPR oximetry is a powerful, minimally invasive technique for measuring tissue oxygen levels, offering greater accuracy and sensitivity than most other methods. Most EPR oximetry approaches exploit the relaxation-enhancing properties of molecular oxygen, which shortens the EPR relaxation times of other paramagnetic species [91–94]. The enhancement of relaxation rates increases proportionally with oxygen concentration across a broad range of oxygen tensions. Measurements based on the T_1_ and T_2_ relaxation times of EPR spin probes introduced into a biological system provide a direct indication of tissue oxygenation status. The most common approach involves measuring the broadening of the EPR linewidth (ΔBpp), which is inversely related to T_2_. In practice, a given paramagnetic material is first calibrated to determine the effect of oxygen on its linewidth. When introduced into tissues, the linewidth of the probe can then be directly interpreted in terms of local oxygenation [91–94]. EPR oximetry has been applied across a wide variety of tissues under both physiological and pathological conditions (reviewed in [95]). However, some paramagnetic materials may interact undesirably with tissues, either causing adverse reactions or losing responsiveness to oxygen. To overcome these limitations, substantial progress has been made in developing biocompatible EPR oxygen sensors, most notably through encapsulation strategies and the design of implantable microdevices [96]. Two classes of paramagnetic compounds are used as EPR oxygen sensors: soluble materials and insoluble particulate materials. For soluble paramagnetic materials, the LW varies linearly with increasing concentration of oxygen [91]. Two types of structures are interesting within the class of soluble oxygen sensors: the nitroxides and the triarylmethyl (or trityl) radicals. The effect of oxygen on the linewidths of these compounds is fairly modest. To increase the sensitivity of the oxygen measurement, strategies have been used to encapsulate soluble oxygen sensors in lipophilic environments. As oxygen is more soluble in lipophilic environments than in water (the concentration of oxygen will be higher in a lipid phase than in a water phase for the same partial pressure of oxygen pO_2_), an increase in sensitivity can be achieved using these systems (Fig. 5). This was first achieved using proteinaceous microspheres filled with nitroxides dissolved in an organic liquid solvent [97]. This strategy has been expanded and applied to trityl radicals to produce sensors highly sensitive to changes in the oxygen environment. As examples of development, we may cite nano-emulsions containing fluorinated trityl radicals dissolved in perfluorocarbons [98, 99], trityl-loaded lipid nanocapsules [100, 101], in-situ-oleogel and emulgel containing lipophilic trityls [102], and nanoscaffold of aluminum hydroxide (boehmite) functionalized with a deuterated Finland trityl radical [103]. Of note, lipid-based carrier systems have also been proposed as stabilized systems including lipophilic iron-dithiocarbamate complexes with a high efficiency for the EPR spin trapping of nitric oxide [104].

**Fig. 6:**
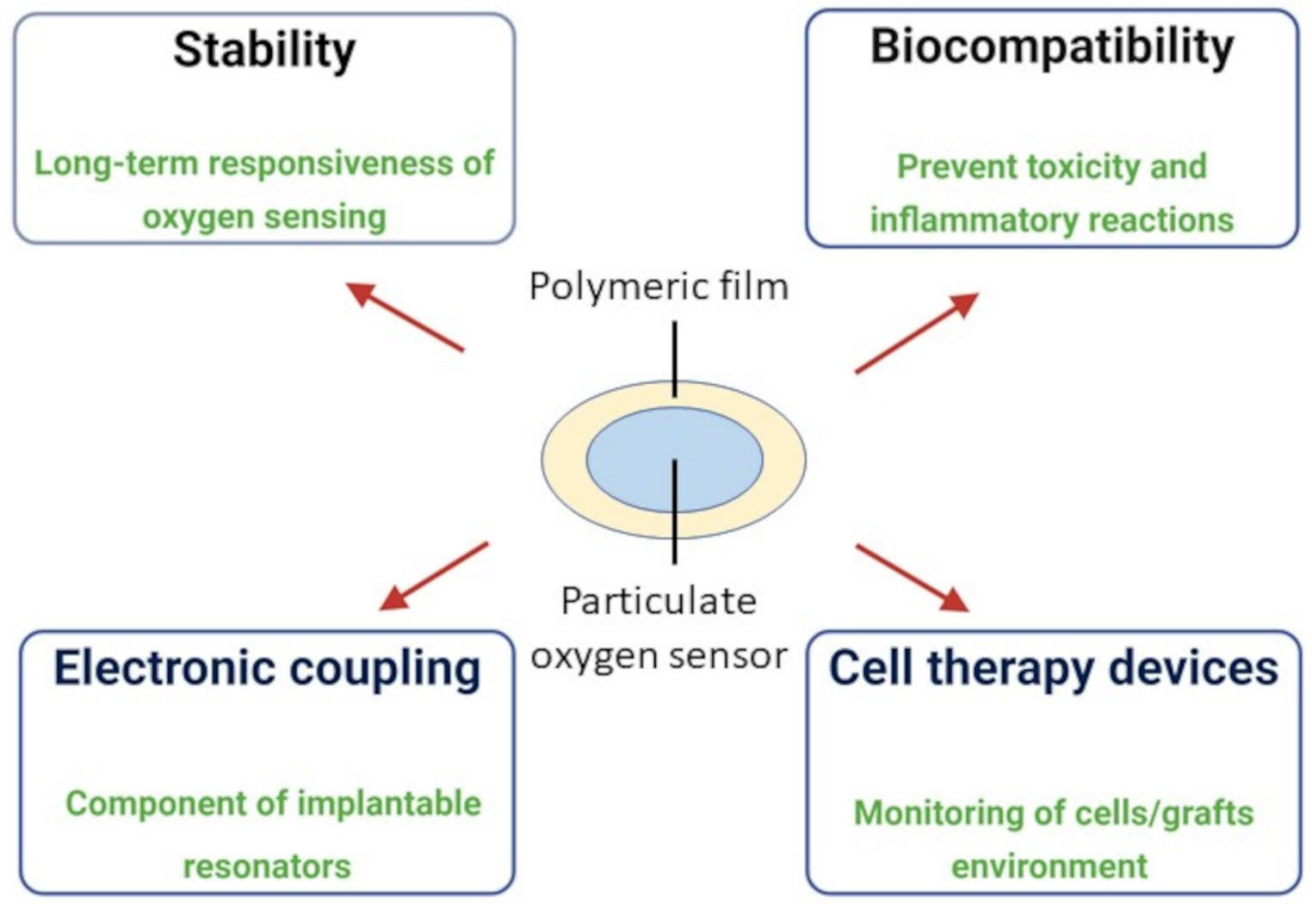
Benefits from the inclusion of particulate EPR oxygen sensors in polymeric films. Encapsulation can also improve the long-term stability of the sensor’s responsiveness to oxygen. It enhances biocompatibility, reducing the risk of toxicity or inflammatory reactions. The incorporation of oxygen sensors into biocompatible matrices has also enabled the development of implantable resonators for measuring oxygen at substantial tissue depths. Incorporating oxygen sensors into cell therapy implantable devices or grafted tissues can serve as an indicator of both oxygenation and vascularization

For solid particulate paramagnetic materials, the relationship between EPR linewidth and oxygen partial pressure is more complex. A major advantage of using particulate materials is that those identified as oxygen sensors exhibit exceptionally large EPR linewidth broadening per unit of pO_2_ (several orders of magnitude greater than that of soluble radicals) [92–94]. Particulate oxygen sensors include carbon-based materials (such as carbon blacks, chars, and charcoals) as well as lithium phthalocyanine and its derivatives. Coating or encapsulating particulate oxygen sensors offers multiple benefits (Fig. 6). By preventing direct contact between the sensor and tissue cells, it enhances biocompatibility, reducing the risk of toxicity or inflammatory reactions [96, 105]. Encapsulation can also improve the long-term stability of the sensor’s responsiveness to oxygen and prevent particle dispersion or migration within tissues. Furthermore, embedding particulate sensors in larger systems such as implantable resonators can provide a structured and protected environment, enabling repeatable, long-term measurements even at deep tissue sites (Fig. 5). Both nano-/micro-encapsulation and macroscopic device formats have been reported for particulate oxygen sensors.

Nanoencapsulation has been employed to prepare charcoal suspensions with long-term stability, as demonstrated for fusinite charcoal particles coated with arabic gum, poloxamer, and polyvinylpyrrolidone polymers [106]. In char particulates that gradually lost oxygen responsiveness in tissues, appropriate surface coatings were shown to preserve the oxygen sensitivity of the paramagnetic material in vivo [107]. Another important class of EPR oxygen sensors is found among carbon blacks, produced by the incomplete combustion or thermal decomposition of aromatic oils or other hydrocarbons [108]. Biocompatible inks containing carbon black particles have been formulated using polymers such as carboxymethyl cellulose (CMC), hydroxypropyl methyl cellulose (HPMC), or polyvinylpyrrolidone (PVP) [109]. These inks were subsequently used in the first clinical EPR studies to measure tissue oxygenation [110, 111].

Larger-sized implants have also been developed, ensuring both sensor biocompatibility and the option for retrieval at the conclusion of a study. The first biocompatible oxygen sensor implants were described for fusinite charcoal particles embedded in a polydimethylsiloxane (PDMS) or silicone matrix [112]. Thin films incorporating paramagnetic materials were prepared with various biopolymers, such as cellulose acetate, cellulose triacetate, cellulose nitrate, silicone, and polyurethane, to enhance their long-term responsiveness in tissues [113]. Lithium phthalocyanine (LiPc) crystals were embedded in thin biocompatible films [114] and in polytetrafluoroethylene (Teflon AF2400) micro-implants [115], providing a stable, inert microenvironment for the oxygen sensor. The same oxygen-permeable polymer has also been used to encapsulate lithium naphthalocyanine (LiNc), ensuring both long-term stability and biocompatibility [116]. Structurally flexible PDMS was cast-molded to fabricate chips (known as OxyChips) containing lithium octa-n-butoxynaphthalocyanine (LiNc-BuO) [117–120]. Encapsulation in PDMS did not alter the oxygen sensitivity of the crystals, and the chips remained stable after sterilization. In vivo oximetry demonstrated that OxyChips could provide reliable, repeated measurements of tissue oxygenation without inducing inflammatory or adverse reactions at the implantation site, confirming their safety and biocompatibility [117–120]. More recent designs incorporate oxygen-sensing probes embedded with gold nanoparticles, enabling X-ray visualization for spatial and anatomical localization of the implant, thereby improving interpretation of oxygen data [121]. These OxyChips have been successfully applied in clinical EPR oximetry studies [122, 123]. In these trials, OxyChips were implanted into patient tumors, and repeated oxygenation measurements were obtained at baseline and following a hyperoxygenation challenge. The results demonstrated the feasibility of individualized tumor oxygenation assessment to guide and optimize clinical interventions, particularly in the context of radiotherapy [122].

The incorporation of oxygen sensors into biocompatible matrices has also enabled the development of implantable resonators, a concept first introduced by Hal Swartz [124]. These systems function as insertable devices for measuring oxygen at substantial tissue depths. In the catheter-resonator approach, for example, a micro-coil is positioned at the end of a very thin wire, with the paramagnetic sensor placed at the center of the loop and held in place by a thin layer of biocompatible polymer [105]. The first implantable resonators were constructed using lithium phthalocyanine embedded in Teflon AF2400 polymer [114], and studies in rabbits demonstrated their ability to detect oxygenation changes deep within tissues [114]. Using the same sensor-polymer combination, larger devices were developed featuring two sets of loops: a large loop at one end for inductive coupling to an L-band EPR spectrometer, and one or more small loops at the other end containing oxygen-sensitive paramagnetic material, which could be implanted at the target sites [125]. These implantable resonators allowed repeated pO_2_ measurements with excellent temporal resolution over days or weeks in organs such as the brain, heart, and muscles [125, 126], as well as in brain tumors [127, 128]. Similarly, resonators built with lithium octa-n-butoxynaphthalocyanine (LiNc-BuO) crystals embedded in PDMS were used to measure oxygen in myocardial tissue and the lung pleura [129, 130]. More recently, a refined version of the implantable resonator incorporated LiNc-BuO crystals in PDMS, with an additional biocompatible polytetrafluoroethylene coating applied over the PDMS-coated transmission lines and circular disk to preserve the shape of the coupling loop [131]. Preclinical studies in rabbits confirmed that this device could be safely implanted in brain and leg tissue, providing repeated, noninvasive EPR oxygen measurements for up to nine months in vivo [131].

An important application at the intersection of EPR oximetry and biomaterials lies in research on transplantation and cell therapy. Tissue grafts and cell therapy devices used in tissue engineering and regenerative medicine require an adequate oxygen supply within a biocompatible environment. To this end, incorporating oxygen sensors into implantable devices or grafted tissues can serve as an indicator of both oxygenation and vascularization. Early developments included the use of skeletal myoblasts [132, 133] and mesenchymal stem cells [134] doped with paramagnetic oxygen sensors, which were engrafted into ischemic or infarcted regions of mouse hearts. Similarly, pO_2_ measurements have been performed to monitor oxygen evolution in ovarian grafts after in vivo implantation [135–137]. Because hypoxia is a major cause of impaired wound healing, nanomedicine and cell therapy approaches are being explored to improve tissue perfusion and oxygenation [138]. EPR oximetry has been used to dynamically monitor oxygenation in wounds and flaps [139–142]. For example, adipose-derived stromal cells seeded onto a human acellular collagen matrix (a biological dressing) have shown promise for treating nonhealing wounds by enhancing dermal angiogenesis and remodeling [142]. Bioartificial pancreas and β-cell replacement therapies are emerging strategies for treating type I diabetes. However, insufficient oxygenation can limit the long-term survival of encapsulated islet grafts. Innovative designs have been developed to ensure graft biocompatibility. In xenograft applications, immune isolation of transplanted cells is essential, while still permitting access to nutrients and oxygen. Strategies include modifying the encapsulation biomaterial and co-encapsulating angiogenesis-promoting cells. In this context, EPR spectroscopy and imaging provide a unique means of monitoring oxygenation within implanted grafts over time [143–149].

### Current challenges and future perspectives: potential clinical integration and interdisciplinary synergies

Innovative approaches are emerging to address unmet clinical needs in oncology, neurodegenerative disorders, and currently incurable chronic diseases. In this context, the next generation of DDSs and devices for cellular therapies is particularly promising, as their smart configurations can be finely tuned to adapt to microenvironmental constraints and release drugs or therapeutic factors on demand, thereby optimizing therapeutic responses. The in vitro and in vivo validation of these highly adaptive systems can greatly benefit from advanced, non-destructive methods capable of simultaneously and dynamically characterizing the evolution of crucial factors within DDSs or biomaterials. In this regard, EPR spectroscopy and imaging offer a wealth of information that can be leveraged by researchers in advanced pharmaceutical technology.

As EPR continues to evolve toward clinical applications [122, 123, 150–153], several key challenges must be addressed. We have already emphasized the importance of ensuring the biocompatibility of implanted devices. While preclinical studies can rely on state-of-the-art materials and formulations, the requirements for application in healthy volunteers or patients may represent a significant hurdle. Importantly, regulations on biocompatibility extend not only to the DDS or biomaterial used as a carrier, but also to the EPR probes employed to label the system. Regulatory approval by health authorities and institutional review boards is expected to be particularly demanding, and further studies are needed to establish the safety of probes such as nitroxides, trityls, and phthalocyanine derivatives. Bridging the translational gap will also require attention to the scalability of production and compliance with Good Manufacturing Practices. Another crucial consideration is the development of EPR spectrometers and imaging systems suitable for patient use, along with resonators specifically adapted for clinical investigations [130–156]. Since EPR analysis in deep tissues requires operating at lower frequencies, which inevitably reduces sensitivity, improving detection sensitivity remains a cornerstone for expanding the scope of clinical EPR applications. In recent decades, significant progress has been made in optimizing detection sensitivity through rapid-scan and multi-harmonic acquisition methods compatible with low-frequency EPR systems [157–165], including clinical spectrometers [166]. Ultimately, the successful clinical translation of EPR-based approaches will rely on a multidisciplinary effort, bringing together expertise from diverse scientific and technological fields.

## Conclusions

EPR has proven to be a versatile and powerful technique for advancing research in both drug delivery systems and biomaterials. By exploiting spin-labeling strategies or the intrinsic paramagnetic properties of specific probes, EPR makes it possible to interrogate structural organization, molecular mobility, and local microenvironments within complex systems. These capabilities allow for real-time assessment of drug release, stability, and degradation processes, as well as quantitative tracking of nanocarriers in theranostic applications. In parallel, EPR has shed light on radical-driven phenomena in biomedical materials, such as those underlying bone cements and dental resins, while the integration of paramagnetic sensors into biocompatible matrices has expanded its utility to monitoring oxygen and nitric oxide in living tissues. Altogether, these contributions highlight EPR not only as a tool for mechanistic understanding but also as a foundation for developing innovative diagnostic and therapeutic platforms in nanomedicine and regenerative medicine.

## Data Availability

Not applicable.
